# Cardiorespiratory Performance, Physical Activity, and Depression in Thai Older Adults with Sarcopenia and No Sarcopenia: A Matched Case-Control Study

**DOI:** 10.3390/ijerph21060724

**Published:** 2024-06-01

**Authors:** Nuntiya Boontanom, Patcharee Kooncumchoo, Kornanong Yuenyongchaiwat

**Affiliations:** 1 Physiotherapy Department, Faculty of Allied Health Sciences, Thammasat University, Pathum Thani 12120, Thailand; 2 Center of Excellence in Creative Engineering Design and Development, Thammasat University, Pathum Thani 12120, Thailand; 3 Thammasat University Research Unit for Physical Therapy in Respiratory and Cardiovascular Systems, Thammasat University, Pathum Thani 12120, Thailand

**Keywords:** sarcopenia, older adults, cardiorespiratory performance, physical activity, depression, matched case–control study

## Abstract

Background: Older adults have a high risk for musculoskeletal, cardiorespiratory, and mental health problems. We compared respiratory muscle strength, cardiovascular endurance, physical activity (PA), and depression between older adults with and without sarcopenia. Methods: This matched case–control study included 200 Thai older adults (100 participants with and without sarcopenia). According to the Asian Working Group for Sarcopenia 2019, participants completed a handgrip dynamometer, a 6 m walk test, and bioimpedance analysis for sarcopenia screening. Individuals were required to evaluate their cardiovascular endurance and respiratory muscle strength and complete a set of questionnaires (i.e., depression and PA). Participants with and without sarcopenia were compared using a *t*-test, and ANOVA was used for subgroup analysis. Results: Participants with sarcopenia had significantly lower inspiratory muscle strength (*p* < 0.001), functional capacity (*p* = 0.032), PA (*p* < 0.001), and higher depression scores (*p* < 0.001) than those without sarcopenia. Respiratory muscle strength and PA were significantly reduced in those with severe sarcopenia, followed by those with sarcopenia, possible sarcopenia, and no sarcopenia. Older adults with severe sarcopenia had higher depression scores than those with sarcopenia, possible sarcopenia, or no sarcopenia. Conclusions: Older adults with sarcopenia may exhibit lower cardiorespiratory performance, less PA, and higher depression than those without sarcopenia.

## 1. Introduction

Typically, the aging process is associated with reduced physical activity (PA) due to loss of muscle and bone mass and a decline in tissue elastic capacities [1]. Quantitative and qualitative changes in skeletal muscle structure and function are known to accompany the aging-related loss of muscular function. The loss in muscle mass and function is considered the most dramatic and substantial of all age-related changes, a condition defined as sarcopenia characterized by poor physical performance or physical strength [2]. Among older adults, sarcopenia has been extensively investigated and debated in public health policies. As the number and proportion of older individuals continue growing in the population, sarcopenia-related morbidity will become an area of increasing healthcare resource utilization. 

Respiratory sarcopenia is a syndrome characterized by muscle fiber atrophy and weakening of the respiratory and systemic skeletal muscles due to advanced age. Respiratory sarcopenia results in a decrease in respiratory force generation and pulmonary function, which negatively impacts daily activities and the quality of life [3]. The deterioration of respiratory muscle strength may have serious clinical consequences. Hypoxia is known to cause reduced oxygenation and multisystem organ failure owing to poor respiratory function. Impaired ventilation has been implicated in enhancing the risk of cardiovascular disease when low exercise capacity is combined with increased oxidative stress [4]. Characteristics of respiratory muscles and their relationship with recognized sarcopenia parameters, including lower lean mass, poor muscular strength, and functional impairment, have been examined in older individuals with sarcopenia [3,4,5]. Respiratory muscle weakness during aging can be attributed to proteolysis and increased collagen synthesis combined with increased rigidity of the chest wall [6]. Furthermore, both age and sarcopenia reportedly impact the diaphragm, the most important respiratory muscle. Among older adults, transdiaphragmatic pressure, a measure of diaphragmatic muscle activity, reportedly reduces by 20–41%, along with a 30% reduction in overall respiratory muscle strength [7]. In human autopsy studies, aging was linked to histopathologically abnormal findings in the diaphragm structure, such as small diaphragm size or the loss of cytoplasmic integrity [3].

Older adults not only experience a reduction in respiratory muscle strength but also a decrease in cardiovascular endurance. Furthermore, cardiorespiratory function is known to be reduced in older individuals. Importantly, older adults exhibit a high risk of developing sarcopenia. Moreover, older adults with physical inactivity or depression are at a risk of developing sarcopenia [8,9,10].

The prevalence of sarcopenia and its relationship with cardiorespiratory performance, physical decline, and depression have been explored previously, although comparisons between individuals with and without sarcopenia may be limited. Therefore, the purpose of the current study was to compare respiratory muscle strength, cardiovascular endurance, PA, and depression among older adults with and without sarcopenia using age-matched controls. 

## 2. Materials and Methods

Herein, we performed a cross-sectional study using an age-matched case–control design to compare respiratory muscle strength, functional capacity, PA, and depression between older adults with and without sarcopenia.

The recruitment of community-dwelling older participants with sarcopenia and without sarcopenia were classified using the Asian Working Group for Sarcopenia 2019 criteria, which included screening muscle strength and/or physical performance plus skeletal muscle mass index (SMI) [11]. Twenty-four hours prior to testing, participants who had a resting heart rate of >120 bpm, severe hypertension (>180/120 mmHg), and uncontrolled diabetes mellitus, high body temperature, respiratory infection (i.e., COVID-19) were excluded. Individuals who used assistive devices, such as canes and walkers, as well as those who had a history of cardiovascular disease, neurological problems, or musculoskeletal pain that affected walking or performing the test, were excluded. 

Muscle strength was evaluated by using TKK digital dynamometer (TKK 5101 Grip-D, Takey, Tokyo Japan). All participants were required to stand with their feet shoulder-width apart and straight arms. The participants were asked to grasp as much as they could hold for 3 s. The maximal grip strength was recorded in two trials, and the average handgrip strength value was used. A cut-off point of <28 kg for males and <18 for females suggested low muscle strength [11].

A 6 m walk test was used to measure gait speed. Two trials were performed, wherein participants were required to walk, and the average gait speed was recorded. Slow gait speed or poor physical performance was defined as <1.0 m/s [11]. 

SMI was assessed using a bioelectrical impedance analyzer (BIA) (Omron KARADA Scan Body Composition & Scale, HBF-375). Participants were categorized as having a low SMI at values of <7 kg/m^2^ in males or 5.7 kg/m^2^ in females [11].

As mentioned previously, participants who met the criteria for slow gait speed and/or low grip strength and SMI were identified as having sarcopenia, whereas those who did not meet these criteria were classified as older adults without sarcopenia. 

Cardiorespiratory performance (i.e., respiratory muscle strength, and functional capacity) was assessed in all participants. A MicroRPM Respiratory Pressure Meter (Micro Medical/CareFusion, Kent, UK) was used to evaluate respiratory muscle strength. Participants were asked to sit on a chair with their feet on the floor, exhale until residual volume was inhaled through the device as fast and hard as possible, and then hold for 1.5 s. The measurements were repeated three times, and the highest maximum inspiratory pressure (MIP) value was selected. Maximum expiratory pressure (MEP) was measured by inhaling until total capacity, exhaling as fast and hard as possible, and then holding for 1.5 s. The measurements were repeated three times, and the highest value was selected [12].

Cardiovascular endurance can be defined as functional capacity, which was assessed using a 6 min walk test (6-MWT) with a 30 m long course in a lane [13]. During the 6 min, all participants were instructed to walk back and forth in a straight line as possible. The total distance walked over 6 min was noted as the 6 min walk distance (6-MWD).

Additionally, a set of questionnaires was used to evaluate depression and physical activity. Depression was assessed using the Thai Geriatric Depression Scale (TGDS), with a total accuracy of 0.93 [14]. The participants were required to rate their depression during the previous week. The self-reported TGDS scores ranged from 0 to 30, with a score of 1–12 indicating no symptoms of depression [14].

The Global Physical Activity Questionnaire (GPAQ), developed by the World Health Organization, is widely used to evaluate PA [15]. The questionnaire comprised three domains: activity at work, travel to and from places, and recreational activities. A high level of PA was defined as ≥1500 metabolic equivalent tasks (MET) min per week, and low PA was defined as less than 600 MET min/week [16].

Data analyses were performed using the SPSS version 26.0 (IBM Corp., Armonk, NY, USA). Descriptive statistics were used to describe the sample characteristics. Normal distribution was tested using the Kolmogorov–Smirnov test. We explored differences in respiratory muscle strength, functional capacity, depression, and PA between participants categorized as having sarcopenia and those without sarcopenia using independent *t*-test analyses for normal distribution. Furthermore, subgroup analyses (severe sarcopenia, sarcopenia, possible sarcopenia, and no sarcopenia) were performed using ANOVA. A *p*-value of <0.05 was deemed statically significant.

## 3. Results

**Figure 1:**
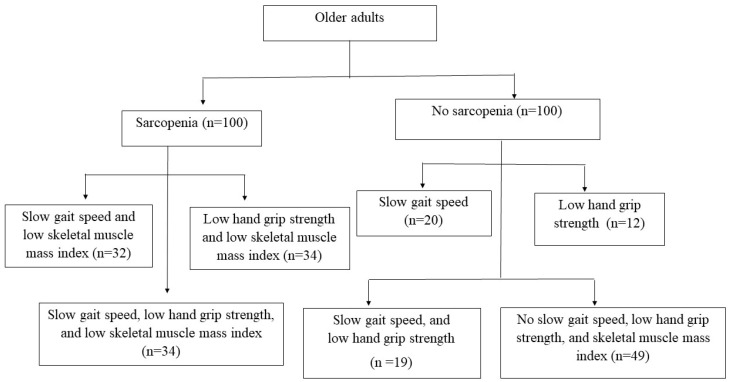
Flow chart of the study.

Using the AWGS 2019 criteria, 200 Thai participants with and without sarcopenia were recruited. Figure 1 presents a flowchart of the sarcopenia assessment conducted among older Thai adults. According to the AWGS criteria 2019, 100 older adults with sarcopenia had low muscle plus slow gait speed (n = 32), low muscle plus low handgrip strength (n = 34), low muscle plus slow gait speed, and low handgrip strength (n = 34). In contrast to those without sarcopenia, 20 older adults had slow gait speed, 12 older adults had low handgrip strength, 19 older adults had both slow gait speed and poor handgrip strength, 23 older adults had neither slow gait speed nor poor handgrip strength, and 23 older adults had only low SMI. A subgroup analysis was performed to compare older adults with sarcopenia (defined as low SMI and low handgrip strength or slow gait speed), severe sarcopenia (defined as low SMI, low handgrip strength and slow gait speed), possible sarcopenia (defined as no low SMI but low handgrip strength or/and slow gait speed), and no sarcopenia (defined as normal SMI, handgrip strength and gait speed). Therefore, the subgroup analyses included 66 older adults with sarcopenia, 34 with severe sarcopenia, 51 with possible sarcopenia, and 49 without sarcopenia. 

Table 1 summarizes the characteristics of older adults in the sarcopenia and non-sarcopenia groups. The average participant age was 72.26 ± 4.28 years. There were no significant differences in sex or medical condition between older adults with and without sarcopenia (*p* > 0.05). Additionally, older adults with sarcopenia had lower inspiratory respiratory muscle strength (MIP; 46.73 ± 21.06 vs. 59.22 ± 26.89, 95% confidence interval (CI): −19.23 to −5.75, *p* < 0.001), lower functional capacity (6-MWD: 309.13 ± 72.51 vs. 332.19 ± 77.18 m, 95% CI: −43.95 to −2.18, *p* = 0.031), lower PA levels (GPAQ: 1266.68 ± 2308.98 vs. 3916.40 ± 6943.68 MET min per week, 95% CI: −4092.75 to −1206.69, *p* < 0.001), and higher depression scores (9.02 ± 6.84 vs. 5.97 ± 4.52, 95% CI: 1.43 to 4.67, *p* < 0.001) than older adults without sarcopenia.

Subgroup analysis was conducted using the AWGS 2019 criteria (i.e., severe sarcopenia, sarcopenia, possible sarcopenia, and no sarcopenia). Low MIP, 6-MWD, and PA levels and high depression scores were observed in older adults with severe sarcopenia, followed by those with sarcopenia, possible sarcopenia, and no sarcopenia. (*p*s < 0.05) (Table 2).

**Figure 2:**
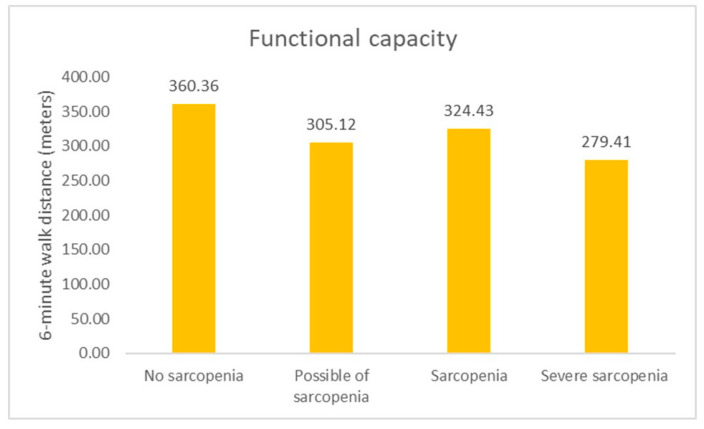
The proportion of participants according to sarcopenia assessed by the sarcopenia and functional capacity (n = 200).

**Figure 3:**
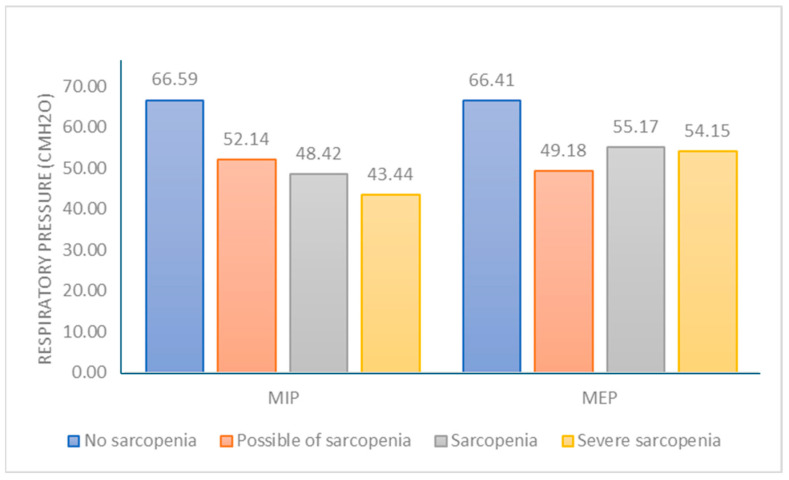
The proportion of participants according to sarcopenia assessed by the sarcopenia and respiratory muscle strength (n = 200).

**Figure 4:**
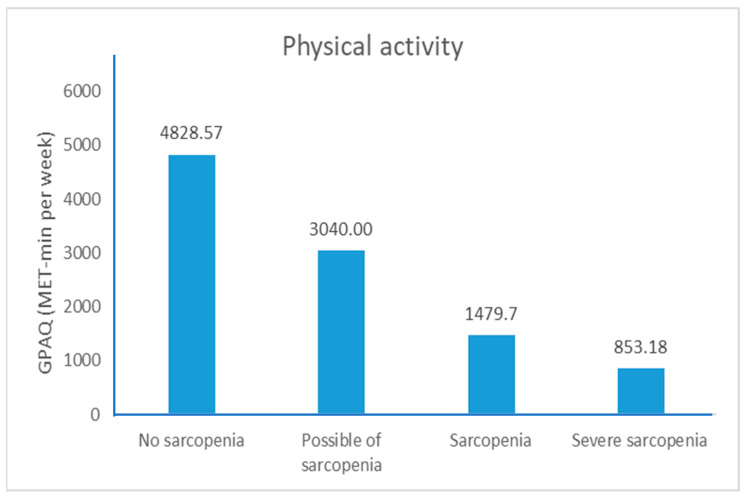
The proportion of participants according to sarcopenia assessed by the subgroup of sarcopenia and GPAQ (n = 200).

**Figure 5:**
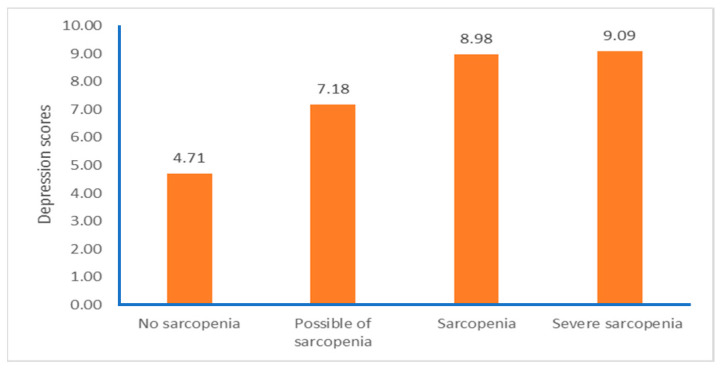
The proportion of participants according to sarcopenia assessed by the sarcopenia and depression scores (n = 200).

A statistically significant difference was detected at the 0.001 distance level, as reported in the 6-MWT. Older adults with severe sarcopenia had the lowest 6-MWD (Figure 2), respiratory muscle strength (Figure 3), PA (Figure 4), and high depression (Figure 5). Furthermore, older adults with possible sarcopenia had lower cardiorespiratory performance (i.e., MIP, MEP, and 6-MWD) than older adults without sarcopenia (*p*s < 0.05).

## 4. Discussion

Herein, we designed an age-matched control group to compare cardiorespiratory performance, PA, and depression scores between 100 older adults with sarcopenia and without sarcopenia, respectively. Our findings confirmed that older adults with sarcopenia had lower cardiorespiratory performance and PA and higher depression scores than those without sarcopenia. Focusing on the severity of sarcopenia, we observed that older adults with severe sarcopenia exhibited the lowest cardiorespiratory performance and PA and the highest depression scores. 

Low inspiratory muscle strength has been reported in older Thai adults with or without sarcopenia. According to the American Thoracic Society (ATS)/European Respiratory Society statement on respiratory muscle testing, an MIP of 80 cmH_2_O indicates clinically significant inspiratory muscle weakening [12]. According to a systematic review of MIP, adults aged 60–69 years had MIP values of 92.7 and 75.1 cmH_2_O in males and females, respectively. Older adults aged 70–83 years had MIP values of 76.2 cmH_2_O in males and 65.3 cmH_2_O in females [17]. In the present study, older adults aged 65–69 years had an MIP of 73.7 cmH_2_O in males (sarcopenia, 64.27 cmH_2_O; no sarcopenia, 78.67 cmH_2_O) and 58.1 cmH_2_O in females (sarcopenia, 42.2 cmH_2_O; no sarcopenia, 60.36 cmH_2_O). For older adults aged 70–80 years, the MIP was 63.4 in males (sarcopenia, 58.14 cmH_2_O; no sarcopenia, 68.21 cmH_2_O) and 41.9 cmH_2_O in females (sarcopenia, 39.21 cmH_2_O; no sarcopenia, 44.75 cmH_2_O). To categorize sarcopenia in older adults, an MIP cut-off of ≤55 and ≤45 cmH_2_O has been established for males and females, respectively [18]. Considering MEP, a cut-off of ≤60 and ≤50 cmH_2_O has been established in males and females, respectively [18]. Accordingly, the older adults with sarcopenia or no sarcopenia in our study displayed lower MIP values than those reported previously. Moreover, we found that the presence of sarcopenia could significantly reduce MIP values. Reduced respiratory muscle strength and decreased muscle mass are related to advanced age and can contribute to the decline in physical performance and muscle function [3,19]. These consequences could be attributed to reduced diaphragm muscle mass and could exacerbate age-related alterations in the lung and chest wall while impairing ventilatory actions [20]. Overall, the presence of muscle fiber atrophy and fiber type-specific weakness, both in higher force- and lower force-generating capacities (i.e., type IIx and/or IIb fibers, and type I and IIa fibers, respectively), has been suggested [21]. Hence, muscle atrophy and reduced contractile force capacity (i.e., loss of type II fibers: hypoplasia) result in muscle weakness and a decline in physical performance. 

The MEP reflects the strength of the abdominal and other expiratory muscles, including those involved in coughing. Nevertheless, no statistically significant differences in MEP were detected in the sarcopenia and non-sarcopenia groups or in male and female participants, in contrast to the findings of Sawaya et al., where MEP was associated with sarcopenia but not MIP values [22]. The authors reported that the rectus abdominis, the expiratory muscle, comprises 46% of type IIB fibers, while the diaphragm muscle, which is the inspiratory muscle, comprises 80% of fatigue-resistant fibers (55% type I and 25% type IIA). In sarcopenia, muscle atrophy was detected in fast-twitch fibers; therefore, expiratory muscle strength was predisposed to reduce in the expiratory muscle, although not in the diaphragm, which showed a small change in muscle mass and muscle strength [22]. Hence, future studies need to explore changes in muscle fibers in the inspiratory and expiratory muscles of older adults with sarcopenia when compared with those in older adults without sarcopenia.

Comparing distances between the sarcopenia and non-sarcopenia groups in the 6-MWT, older adults with sarcopenia exhibited reduced walking for 6 min, indicating that older adults with sarcopenia have a lower functional capacity than those without sarcopenia. Moreover, older Thai adults were found to exhibit a shorter 6-MWD than healthy older individuals in a meta-analysis [23], where the 6-MWD aged 60–69 years was 505–560, and 490–530 m in those aged 70–79 years. In the present study, the 6-MWD was 322–366 m, aged 60–69 years, and 306–322 m aged 70–79 years old, respectively. Decreased leg strength has been observed in individuals who have experienced loss of muscle mass, which may explain why individuals with reduced muscle mass display weakness in their leg muscles. A relationship between muscle strength and sarcopenia has been reported, in which knee extensor and hip abductor muscle strengths reportedly reduced walking distance [24]; however, the study did not assess muscle strength in the quadriceps or lower limb muscles. Therefore, muscle exercises should be explored as potential strategies for sarcopenia.

In this matched case–control study, we found that older adults with sarcopenia had lower PA than those without sarcopenia. Several cross-sectional studies have reported that older individuals with physical inactivity have an increased risk of increased sarcopenia [25,26,27]. A systematic review and meta-analysis of 68 cross-sectional studies comprising 98,502 participants with 18 studies revealed that low PA was a high-risk factor for sarcopenia (Odds ratio = 1.73) [27]. It should be noted that PA plays an important role in preventing and reversing sarcopenia. In a systematic review and meta-analysis of 37 randomized control trials, exercise was found to substantially improve muscle mass, muscle strength, and physical performance [28]. This, in turn, increased mitochondrial capacity and was related to muscle quality, muscle performance, and physical function [29].

Regarding depressive symptoms, we found that older adults with sarcopenia had higher depression scores than those without sarcopenia. In two systematic reviews and meta-analysis studies, sarcopenia was associated with a high prevalence of depression. Additionally, sarcopenia was independently associated with depression (odds ratio, 1.57–1.82) [9,30]. In sarcopenia, depressive symptoms could be attributed to elevated levels of inflammatory mediators (i.e., tumor necrosis factor-alpha, C-reactive protein, and interleukin-6) [31,32,33], capable of increasing interstitial concentrations of norepinephrine, dopamine, serotonin, all neurotransmitters that function in the hypothalamus and hippocampus [30], or elevating inflammation and microglial activation in the hippocampus and frontal cortex, well known to be related to increased anhedonic and depressive-like behaviors [32]. Alternatively, sarcopenia may adversely affect depression through metabolism, endocrine systems, and chronic neuroendocrine immune inflammation [34]. 

This study has some limitations. First, we designed age-matched controls; however, sex-matched control data were collected in a single area, which may not be applicable to the entire population. Second, based on the mechanism of sarcopenia, the study did not assess the strength of the lower limb muscles, which may be another factor affecting functional capacity. Further studies are needed to explore the mechanisms and physiological changes involved in the relationship between sarcopenia and cardiorespiratory performance.

## 5. Conclusions

Our findings demonstrate that older adults with sarcopenia are at risk of adverse health outcomes (i.e., poor cardiorespiratory performance, physical inactivity, and depressive symptoms) when compared with those without sarcopenia. Therefore, these results may be beneficial in developing programs to prevent or reverse sarcopenia in older adults.

## Figures and Tables

**Table 1:** Characteristics of the study.

	Total (n = 200)	Sarcopenia (n = 100)	Non-Sarcopenia (n = 100)	X^2^	*p*-Value
Sex				3.026	0.111
-Male (%)	78 (100)	33 (42.31)	45 (57.69)		
-Female (%)	122 (100)	67 (54.92)	55 (45.08)		
DM (%)	53 (100)	33 (62.26)	20 (37.74)	4.338	0.037
DLP (%)	82 (100)	44 (53.66)	38 (46.34)	0.774	0.472
HT (%)	115 (100)	60 (52.17)	55 (47.83)	0.512	0.562
	**Mean ± SD**	**Mean ± SD**	**Mean ± SD**	**t**	***p*-Value**
Age (years)	72.26 ± 4.28	72.59 ± 4.38	71.92 ± 4.17	1.107	0.269
BMI (kg/m^2^)	23.51 ± 4.15	21.99 ± 3.47	25.04 ± 4.24	−5.565	<0.001
GS (m/s)	0.98 ± 0.20	0.94 ± 0.18	1.02 ± 0.20	−2.785	0.006
HG (kg)	22.02 ± 6.65	19.82 ± 5.25	24.22 ± 7.19	−4.940	<0.001
SMI (kg/m^2^)	6.34 ± 2.27	5.06 ± 0.82	7.61 ± 2.53	−9.590	<0.001
MIP (cmH_2_O)	52.98 ± 24.89	46.73 ± 21.06	59.22 ± 26.89	−3.657	<0.001
MEP (cmH_2_O)	56.22 ± 23.64	54.82 ± 25.17	57.62 ± 22.04	−0.837	0.404
6-MWD (m)	320.66 ± 75.58	309.13 ± 72.51	332.19 ± 77.18	−2.178	0.031
GPAQ (MET min per week)	2591.54 ± 5329.41	1266.68 ± 2308.98	3916.40 ± 6943.68	−3.621	<0.001
Depression (scores)	7.50 ± 5.98	9.02 ± 6.84	5.97 ± 4.52	3.719	<0.001

DM: diabetes mellitus, DLP: dyslipidemia, HT: hypertension, BMI: body mass index, GS: gait speed, HG: handgrips, SMI: skeletal muscle mass index, MIP: maximal inspiratory pressure, MEP: maximal expiratory pressure, 6-MWD: 6 min walk distance.

**Table 2:** Subgroup analysis comparison between no sarcopenia, possible sarcopenia, sarcopenia and severe sarcopenia in cardio-respiratory performance, depression and physical activity among older Thai people.

	NS (n = 49)	PS (n = 51)	SP (n = 66)	SS (n = 34)	*p*-Value NS vs. PS	*p*-Value NS vs. SP	*p*-Value NS vs. SS	*p*-Value PS vs. SP	*p*-Value PS vs. SS	*p*-Value SP vs. SS
MIP (cmH_2_O)	66.59 ± 29.52	52.14 ± 22.15	48.42 ± 19.28	43.44 ± 24.13	0.015	<0.001	<0.001	1.000	0.591	1.000
MEP (cmH_2_O)	66.41 ± 26.38	49.18 ± 12.04	55.17 ± 21.48	54.15 ± 31.49	0.001	0.061	0.106	0.980	1.000	1.000
6-MWD (m)	360.36 ± 68.48	305.12 ± 75.94	324.43 ± 60.24	279.41 ± 85.11	0.001	0.048	<0.001	0.878	0.621	0.018
Depression (scores)	4.71 ± 3.52	7.18 ± 5.05	8.98 ± 6.74	9.09 ± 7.15	0.203	0.001	0.005	0.563	0.813	1.000
GPAQ (MET min per week)	4828.57 ± 7948.49	3040.00 ± 5763.25	1479.70 ± 2661.03	853.18 ± 1334.06	0.507	0.004	0.004	0.637	0.341	1.000

NS: no sarcopenia, PS: possible of sarcopenia, SP: sarcopenia, SS: severe sarcopenia, MIP: maximal inspiratory pressure, MEP: maximal expiratory pressure, 6-MWD: 6 min walk distance, GPAQ: Global Physical Activity Questionnaire.

## Data Availability

Data available on request from the authors.
